# Higher incidence of trocar site hernia with gallbladder extraction via umbilical versus epigastric trocar port: a multicentre retrospective analysis of laparoscopic cholecystectomy

**DOI:** 10.1007/s00423-025-03721-9

**Published:** 2025-05-08

**Authors:** Eduard A. van Bodegraven, Paulieke C. Oosterwijk, Sanne M. van Aalten, Boudewijn E. Schaafsma, Robert M. Smeenk

**Affiliations:** 1 Department of Surgery, Albert Schweitzer Ziekenhuis, Albert Schweitzerplaats 25, Dordrecht, 3300 AK The Netherlands; 2 Amsterdam UMC, Department of Surgery, Amsterdam, Netherlands; 3 Department of Surgery, Groene Hart Ziekenhuis, Gouda, The Netherlands

**Keywords:** Laparoscopic cholecystectomy, Trocar site hernia, Umbilical trocar port, Epigastric trocar port, Gallbladder extraction

## Abstract

**Background:**

Trocar site hernia (TSH) is a known complication of laparoscopic cholecystectomy (LC). Gallbladder extraction is typically performed through the umbilical or epigastric trocar port. However, data on the incidence of TSH in relation to the extraction site is limited. This study aimed to evaluate the need for surgical repair of TSH following gallbladder extraction through the umbilical versus epigastric trocar port.

**Methods:**

A retrospective cohort study was conducted across two Dutch general hospitals. It assessed the occurrence of TSH after LC and examined commonly described risk factors in relation to the TSH location.

**Results:**

Among 2 377 patients that underwent LC, the extraction site of the gallbladder was known in 1756 patients. Gallbladder extraction was performed via the umbilical trocar port in 929 (53%) of cases and via the epigastric trocar port in 827 (47%) of cases. TSH repair was required in 36 (2.1%) patients, with a higher incidence in patients with gallbladder extraction through the umbilical trocar port (3.2%) compared to the epigastric trocar port (0.7%), (*p* < 0.001).

**Conclusion:**

The need for operative repair of a TSH after a LC is significantly reduced when the gallbladder is retrieved through the epigastric trocar site port compared to the umbilical trocar site port.

## Introduction

Since the early 1990’s laparoscopic cholecystectomy (LC) has replaced the open technique as golden standard for cholecystectomy [1, 2]. There are great benefits in the laparoscopic approach compared to the open technique, particularly in terms of postoperative pain, length of hospital-stay, large subcostal incisional hernias and time to social participation following surgery [2]. Although the rate of specific complications associated to LC is minimal compared to open surgery, there are several complications specifically related to the laparoscopic part such as the trocar site hernia (TSH) [3, 4]. The TSH was first described in the late 1960s by Fear et al. in gynaecologic surgery [5]. In recent years, there has been variability in prevalence ranging from 0% up to 5.1% and these rates are believed to be underestimated [6–8].

Risk factors for developing TSH can be divided into patient-related factors and surgery related factors, also described as technical factors [7, 8]. Patient factors include diabetes mellitus, age above 70 years and obesity [2, 7, 8]. Well known surgery related factors are the location, trocar-size, closure of fascial layers, and the occurrence of postoperative trocar site infections [2, 6, 7]. TSH primarily occur at the umbilical trocar port (UTP) because this is often a 10- or 12-millimetre (mm) site [2, 9]. Moreover, the midline in the umbilical region is also considered a weaker spot, with the linea alba not being supported by muscle compared to the lateral abdominal wall [1, 10]. The abdominal wall’s vulnerability at the umbilical area is highlighted by the fact that many primary ventral hernias occur around the umbilicus [11].

Although there are several techniques, one of the most standard used laparoscopic trocar positions for cholecystectomy is by two 10–12 mm trocar ports and two five mm trocar ports. One 10–12 mm port is the UTP placed at, or above in obese patients, the umbilicus for the camera and the other is the epigastric trocar port (ETP) in the epigastrium as working port [12]. Extraction of the gallbladder in a retrieval pouch will be through either the UTP or the ETP [13, 14]. Often the fascia at the port of extraction needs to be widened in case of large stones or large gallbladder. The location of extraction of the gallbladder depends on the surgeon’s preference. Previous studies show that retrieval though the UTP may be associated with less postoperative pain and shorter operation time [15]. Evidence on the occurrence of TSH after LC is scarce, especially in relation to the extraction site of the gallbladder [16].

This study aimed to assess the need for surgical repair of TSH after LC in relation to the extraction site of the gallbladder, in a large retrospective cohort in two Dutch general hospitals.

## Methods

### Design

This retrospective cohort study analysed data from all patients who underwent a LC between 01-01-2013 and 31-12-2015 in two medical centres in the Netherlands: Albert Schweitzer Hospital (ASH) and Groene Hart Hospital (GHH). Ethical approval was retrieved by the METC (Ethical committee) Amsterdam University Medical Centre (Amsterdam UMC) and defined as a non WMO (W21_528 #21.584) study. The surgical technique in both centres was the standard technique for a LC [1, 12]. A pneumoperitoneum is created either by the classic closed technique, using Veress needle insertion or by open technique [17]. After placement of the 10–12 mm UTP, with the videoscope in place, the 10–12 mm ETP is positioned followed by two 5 mm trocars in the right lumbar region and right hypochondrium. After dissection, the exposure of the critical view of safety is achieved. Next, the cystic duct and artery can be exposed, isolated, clipped and transected. Finally, the gallbladder is extracted from the liver bed with electrocauterization and removed from the abdomen in a specimen pouch. Patients can be discharged either on the day of surgery or within one day postoperatively.

### Study participants

All consecutive patients who underwent a LC for all indications in ASH or GHH between 1-1-2013 and 31-12-2015 were included. Patients younger than 18 years and patients who required resection of adjacent organs were excluded. Informed consent was waived because of the retrospective character of the study, as the effort to ascertain all informed consents in such a large population was not deemed feasible and this study has no negative impact on the included patients.

### Data-extraction

The data of the patients was retrieved through a search of treatment codes in the electronic patient database of all operated patients in both centres. Retrieved data regarding patient specific factors, surgery specific factors and follow-up is described in Appendix 1. Postoperative radiologic examination was analysed for the radiologic occurrence of TSH. Data retrieval was performed at least 5 years after LC, resulting in a minimal follow-up of five years.

### Outcomes

The primary outcome was the surgical correction of TSH after LC, comparing gallbladder removal through the UTP versus ETP. Secondary outcomes included the overall occurrence of TSH, the correlation between TSH occurrence and the site of removal and associated risk factors. The presence of TSH was diagnosed by clinical examination or confirmation by imaging.

### Statistical analysis

The sample means and standard deviations were computed for the continuous descriptive characteristics, and the count and proportions were calculated for the discrete descriptive characteristics. Clinicopathologic characteristics were compared between patients with UTP and ETP using χ2 statistics for categorical and Wilcoxon testing for continuous variables. Univariate analysis using logistic regression, identified clinically significant factors associated with development of a TSH and were expressed as odds ratios. Multiple variable models were created to evaluate the risk associated with UTP or ETP.

## Results

### Patient characteristics

Between 2013 and 2015 a total of 2 377 patients underwent a LC. Patient characteristics are presented in Table 1, the mean age was 50.46 years [SD 16.11] and 28.2% were male. Indication for a LC was the occurrence of symptomatic gallstones in 2044 patients (86.0%), acute cholecystitis in 284 (11.9%) and gallbladder polyps in 49 (2.1%) patients. In three patients (0.1%) an adenocarcinoma was found. The introduction of the LC was 1 700 (71.51%) times performed through open introduction, 579 (24.36%) times with a Veress needle and not described in 98 cases (4.12%). Conversion to open procedure was needed in in 12 (0.50%). Closure of the fasciae was described in 2 136 (89.86%) patients. The gallbladder was extracted from the UTP in 929 patients (39.1%) and the ETP in 827 (34.8%) patients. In 621 (26.1%) patients extraction site was not described, therefore they were excluded from further data analysis.

**Table 1:** Patient characteristics

Factor	Specification	
Sex (n; %)	Female Male	1 706 (71.8%) 671 (28.2%)
Age (years; [SD])		50.46 [16.11]
Indication for cholecystectomy (n; %)	Symptomatic gallstones^1^	2 044 (86.0%)
	Acute cholecystitis	284 (11.9%)
	Gallbladder polyps	49 (2.1%)
	Adenocarcinoma	3 (0.1%)
Introduction (n; %)	Open	1 700 (71.51%)
	Veress needle	579 (24.36%)
	Not described	98 (4.12%)
Conversion (n; %)		12 (0.50%)
Fascia (n; %)	Closed	2 136 (89.86%)
	Not closed	14 (0.59%)
	Not described	227 (9.55%)
Extraction Site (n; %)	Umbilical trocar port	929 (39.1%)
	Epigastric trocar port	827 (34.8%)
	Not described	621 (26.1%)

1 Defined as: any complaints or symptoms caused by gallstones without signs of acute cholecystitis in the preoperative phase

### Occurrence of TSH

The primary analysis was done with 1 756 patients included. Surgical repair of a TSH was performed in 36 (2.1%) patients. In 30 out of 929 (3.2%) patients repair of the TSH needed after gallbladder extraction at the UTP and in six out of 827 (0.7%) patients after gallbladder extraction at the ETP (Table 2), p-value < 0.001. In total, 90 patients (5.1%) presented after a LC with a TSH confirmed by imaging or clinical evaluation, which could also be asymptomatic. TSH was located in 66 out of 929 patients (7.1%) following gallbladder extraction at the UTP, and in 24 out of 827 patients (2.9%) following gallbladder extraction at the ETP, p-value < 0.001.

**Table 2:** Occurrence of TSH

	Extraction site gallbladder			
	UTP *n* = 929	ETP *n* = 827	Total *n* = 1 756	
TSH with operative hernia repair n (%)	30 (3.2%)	6 (0.7%)	36 (2.1%)	*p* < 0.001
Clinical and/or radiologic observed TSH n (%)	66 (7.1%)	24 (2.9%)	90 (5.1%)	*p* < 0.001

Trocar Site Hernia (TSH), Umbilical Trocar Port (UTP), Epigastric Trocar Port (ETP)

### Secondary outcomes

Table 3 shows the univariate analysis between patients with TSH and those without TSH. Age and the American Society of Anaesthesiologists Physical State (ASA) scores were significantly different in the two groups, with a higher age and higher ASA score in patients with a TSH (respectively *p* < 0.004 and *p* < 0.047). There was no significant difference between TSH and sex or Body Mass Index (BMI). A multi-variable analysis (Table 4) was conducted and showed that ETP (OR 0.211; 95% CI 0.074–0.530) was a statistical significant protective factor for the occurrence of TSH. ASA Score of 2 or higher was a risk factor for TSH (OR 2.092; 95% CI 1.257–3.479). There was no significant difference between sex, Body Mass Index (BMI) or acute cholecystitis as an indication in multivariate analysis.

**Table 3:** Univariate analysis of TSH with operative hernia repair versus non-TSH

	TSH *n* = 36	No TSH *n* = 1 720	*p*-value
Female n (%)	26 (72.2%)	1261 (73.3%)	0.426
Age [mean, SD]	55.9 [16]	50.1 [16]	< 0.001
ASA^2^ ≥ 2	26 (72.2%)	916 (53.2%)	0.004
BMI^1^ mean [SD]	30.1 [6]	28.9 [5]	0.217
Indication Acute cholecystitis n (%)	9 (25.0%)	216 (12.6%)	0.016
Open introduction n (%)	30 (83.3%)	1244 (72.3%)	0.242
Not closed fasciae [mean, SD]	2 (5.6%)	12 (0.7%)	0.101
*Extraction Site*			< 0.001
UTP n (%) ETP n (%)	30 (83.3%) 6 (16.7%)	899 (51.8%) 821 (48.2%)	

1 American Society of Anaesthesiologists Physical State (ASA) Score

2 BMI = Body Mass Index

Variables with missing data were excluded from this analysis, percentages are based on this

**Table 4:** Multivariable analysis for TSH with operative hernia repair

	Odds ratio [95%CI]	*p*-value
ASA ≥ 2	2.092 [1.257–3.479]	0.020
ETP	0.211 [0.084–0.530]	< 0.001

## Discussion

Post operative hernia at the site of the trocar is a common complication of LC [2, 18]. This study shows that removal of the gallbladder trough the trocar opening at the epigastric region results in significant less surgery for post operative hernias compared to extraction of the gallbladder trough the umbilical port. The reason for the reduction of post operative hernias after gallbladder extraction through the ETP could be that the ETP is situated in the upper region of the abdomen, slightly right from the falciform ligament. In this location, the abdominal wall is shielded by the liver, omentum, and the falciform ligament from the inside [19].

Previous studies, as shown in a meta-analysis from 2019, yielded insufficient evidence for a significant difference in the occurrence of TSH after gallbladder extraction through the UTP or ETP [15]. In 2017, a retrospective data analysis reported a 0.94% occurrence of TSH, with all cases occurring at the UTP [20]. A more recent meta-analysis from 2020 showed gallbladder extraction through the ETP was associated with a reduced risk for TSH, though this was not statistically significant [21, 22]. Kulkarni et al. described in their systematic review an association with a significant higher risk of developing TSH after gallbladder extraction through the UTP (RR 2.68, 95%CI:1.06–6.80, *p* = 0.04), favouring the extraction through the ETP [23]. Furthermore, most of these studies focus on post-operative pain within a short period, of typically 24 h to a few days after surgery. Our current study, with a large cohort from the Netherlands, confirms previous data.

Strengths of this study are the large cohort, conducted across two general hospitals with arms divided in a natural experience matter. Also, this small adjustment in surgical practice has a modest reduction in risk, however, it could be beneficial for a large number of patients since LC is performed over a million times annually worldwide. A first limitation of this study is its retrospective design, which precluded standardised long term standard long-term follow-up after LC to diagnose TSH. Studies that structurally include asymptomatic herniation often report a higher incidence of TSH [24]. The incidence of TSH is expected to be higher if patients are not lost to follow-up and if imaging modalities are used to investigate ambiguous clinical findings. Though the need for surgical repair of the hernia, as we used for our primary endpoint analysis, provides us with the more clinically relevant data. In addition, in 25.5% of the patients the site of extraction was not documented, and they were excluded for analysis. Secondly, potential risk factors such as BMI, trocar type and fascia closure technique were not analysed since they were not available in the patient records. Thirdly, the recommendation of epigastric gall bladder retrieval has a potential esthetical downside, since an enlarged incision is more visible epigastric than umbilically.

For future efforts, increased attention to the technical aspects of the trocar insertion might contribute to further reduce TSH rates. Several former studies advise to remove the trocar under vision and to close the fascia of the trocar sites wider than 10 mm, as most of the TSH occur at the 10–12 mm trocar ports [25–28]. In this study the operation reports reported closure of the fascia in 89.9%, in 9.55% it was unknown and in 0.59% it was not closed. Although not statistically significant (*p* = 0.054), there is a trend suggesting that not closing the fascia may not increase the risk of TSH when comparing the TSH and non-TSH groups. Recent studies fund no significant difference between open or closed fascia ports but indicate that the shape of the trocars, such as bladed trocars, and angle of insertion affects TSH rates [29, 30].

Our findings confirm that the need for operative repair of TSH after a LC is significantly reduced when the gallbladder is retrieved through the ETP compared to retrieval through the UTP. This relatively minor adjustment exerts a considerable impact on the incidence of TSH and the necessity for surgical intervention. Consequently, we recommend gallbladder extraction through the ETP.

## Data Availability

No datasets were generated or analysed during the current study.

## Appendix 1

**Table 1:** Retrieved data regarding patient-specific factors, surgery-specific factors, and follow-up:

Category	Factors	Factor specification
Patient specific	Age (Years)	> 18
	ASA score^1^	1–4
	BMI^2^ (kg/m)	
	History of smoking	Yes
		No
	Presents of Diabetes-Mellitus	Present
		Not present
	History of previous abdominal surgery	Yes
		No
Surgery specific	Indication for surgery	Gallstone disease
		Cholecystitis
		Others
	Introduction	Veress Needle
		Open Technique
	Extraction side	UTP
		ETP
	Closure of the Fasciae	Yes
		No
Follow-up	Occurrence of TSH	UTP
		ETP
		Others
	Operative hernia repair	Yes
		No

1. American Society of Anaesthesiologists Physical State (ASA) Score

2. Body Mass Index

## Abbreviations

ASA: American Society of Anaesthesiologists Physical State
ASH: Albert Schweitzer Hospital, Dordrecht
BMI: Body Mass Index
ETP: Epigastric Trocar Port
GHH: Groene Hart Hospital, Gouda
LC: Laparoscopic Cholecystectomy
UTP: Umbilical Trocar Port
TSH: Trocar Site Hernia
